# Outcomes of Patients With a Small and Large Aortic Annulus Following Balloon-Expandable Transcatheter Aortic Valve Replacement Across Flow-Gradient Patterns

**DOI:** 10.1016/j.shj.2025.100456

**Published:** 2025-03-21

**Authors:** Besir Besir, Shivabalan Kathavarayan Ramu, Tamari Lomaia, Maryam Muhammad Ali Majeed-Saidan, Judah Rajendran, Issam Motairek, Serge C. Harb, Rhonda Miyasaka, Grant W. Reed, Rishi Puri, James J.Y. Yun, Amar Krishnaswamy, Samir R. Kapadia

**Affiliations:** a Department of Cardiovascular Medicine, Heart, Vascular and Thoracic Institute, Cleveland Clinic, Cleveland, Ohio, USA; b Department of Internal Medicine, Cleveland Clinic, Cleveland, Ohio, USA; c Section of Cardiovascular Imaging, Department of Cardiovascular Medicine, Cleveland Clinic, Cleveland, Ohio, USA; d Department of Thoracic and Cardiovascular Surgery, Heart, Vascular and Thoracic Institute, Cleveland Clinic, Cleveland, Ohio, USA

**Keywords:** Aortic stenosis, Balloon-expandable valve, High-gradient, Low-gradient, Small annulus, Transcatheter aortic valve replacement (TAVR)

## Abstract

**Background:**

Patients with small annuli are at risk for worse hemodynamic performance after transcatheter aortic valve replacement (TAVR). It is debatable whether a small annulus confers worse outcomes. This study explored the clinical outcomes following TAVR for patients with small and large annuli across flow-gradient subgroups of aortic stenosis (AS).

**Methods:**

This is a retrospective cohort of patients >18 years who underwent TAVR at Cleveland Clinic between 2016 and 2020. Patients were classified into 2 groups according to annular size: small (area ≤430 mm^2^) and large (area >430 mm^2^). Patients undergoing TAVR with self-expanding valves and those with annular sizing using transesophageal echocardiography were excluded. Each group was subclassified into classical low-flow low-gradient (LFLG) AS, paradoxical LFLG AS, normal-flow low-gradient AS, and high-gradient AS. Clinical outcomes included mortality and heart failure rehospitalization.

**Results:**

The study included 1866 patients, of which 709 (38%) had small annuli. There was no difference in heart failure rehospitalization and mortality between the groups in any of the 4 flow-gradient patterns: hazard ratio (HR) ​= ​0.93 (95% confidence interval [CI]: 0.51-1.69) for patients with classical LFLG AS, HR ​= ​0.95, CI (0.62-1.47) for patients with paradoxical LFLG AS, HR = ​1.16, CI (0.49-2.74) for patients with normal-flow low-gradient AS, and HR = ​0.73, CI (0.50-1.07) for patients with high-gradient AS, using large annulus as a reference. Patients with small annuli had higher mean gradients, lower dimensionless valve index, and a higher incidence of hypoattenuated leaflet thickening and structural valve deterioration post-TAVR.

**Conclusions:**

Patients with small and large annuli have similar intermediate-term clinical outcomes post-TAVR across all flow-gradient patterns treated with balloon-expandable valve.

## Highlights

• Patients with a small annulus had similar intermediate-term clinical outcomes post-transcatheter aortic valve replacement compared to those with a large annulus.
• After stratifying by the flow-gradient subtype of aortic stenosis, patients with smaller annuli still had similar intermediate-term clinical outcomes compared to those with larger annuli.
• Patients with a small annulus had higher mean gradients, lower dimensionless valve index, and a higher incidence of hypo-attenuated leaflet thickening and structural valve deterioration post-transcatheter aortic valve replacement than those with larger annuli.

## Introduction

Studies on surgical aortic valve (AV) replacement demonstrated that smaller valve sizes correlate with worse survival and less valve durability at the long-term follow-up.^1^^,^^2^ In addition, studies on the outcomes following transcatheter aortic valve replacement (TAVR) for patients with different flow-gradient patterns of aortic stenosis (AS) showed that patients with classical low-flow low-gradient (C-LFLG) AS have worse survival than those with high-gradient (HG) AS.^3^, ^4^, ^5^, ^6^, ^7^, ^8^

The success of TAVR is affected by multiple factors including the patient’s risk factors and comorbidities, flow-gradient subgroup, valve choice, anatomic factors, and implantation technique. Much importance has been recently given to the aortic annulus size as one of the anatomic factors that could have prognostic implications.^9^, ^10^, ^11^ Studies were conducted to compare outcomes in patients with small and large aortic annuli. Mixed findings have been reported regarding clinical outcomes and valve hemodynamics post-AV replacement, with some studies reporting a greater risk for impaired valve hemodynamics, including higher gradients and impaired prosthesis durability, in patients with a small annulus.^12^, ^13^, ^14^

Given that both the annular size and the flow-gradient pattern could impact clinical outcomes post-TAVR, the present study was conducted to compare the post-TAVR intermediate-term clinical outcomes for patients with a small annulus to those with a large annulus in each of the flow-gradient subgroups of AS.

## Methods

### Study Design

**Figure 1:**
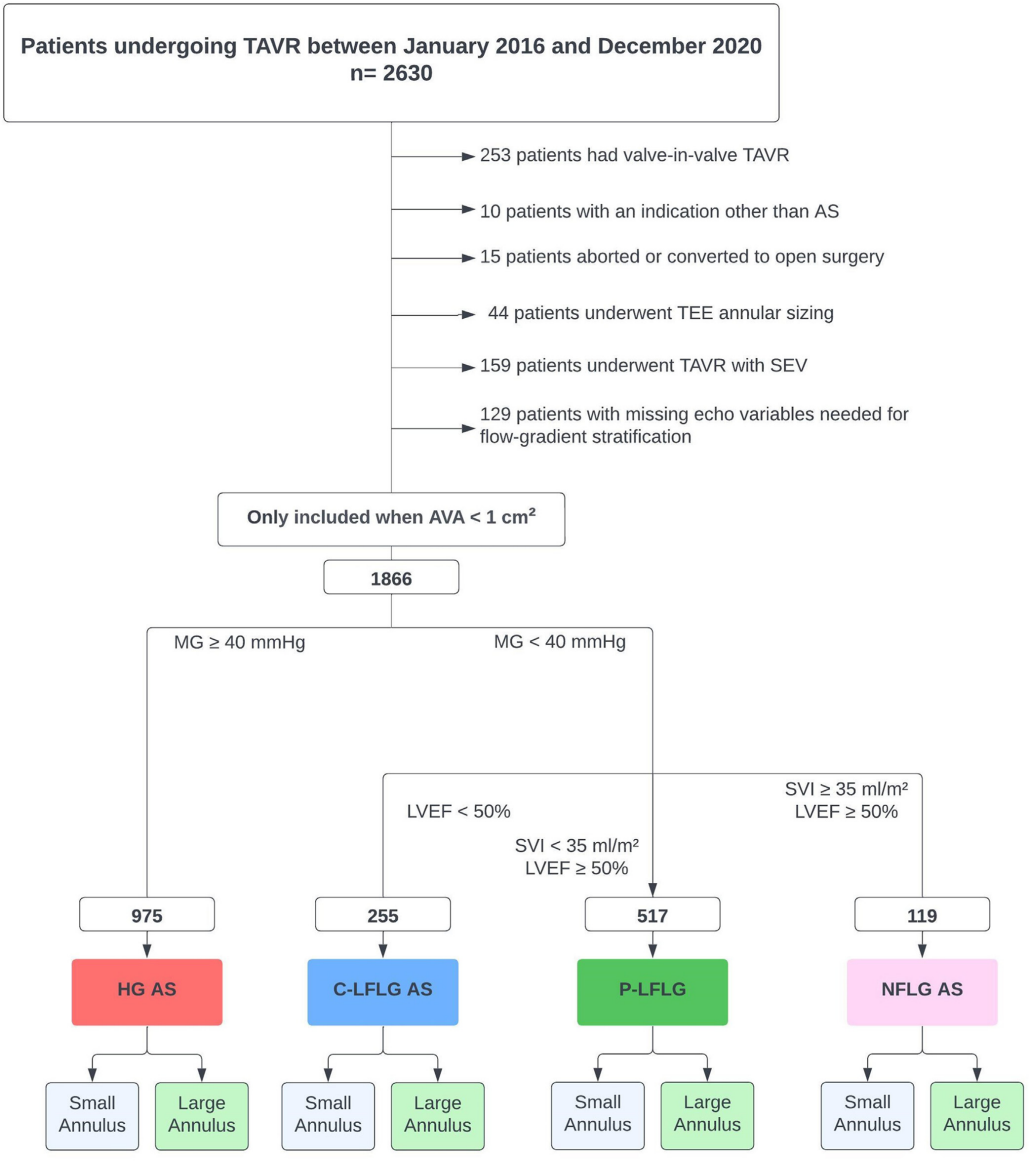
Consort diagram showing the exclusion criteria of the study. Abbreviations: AS, aortic stenosis; AVA, aortic valve area; C-LFLG AS, classical low-flow low-gradient aortic stenosis; HG AS, high-gradient aortic stenosis; LVEF, left ventricular ejection fraction; MG, mean gradient; NFLG AS, normal-flow low-gradient aortic stenosis; P-LFLG, paradoxical low-flow low-gradient; SEV, self-expanding valve; SVI, stroke volume index; TAVR, transcatheter aortic valve replacement; TEE, transesophageal echocardiography.

This study is a single-center, retrospective analysis of data from the Cleveland Clinic database. The study was approved by the institutional review board. From January 2016 until December 2020, a total of 2630 patients underwent TAVR. The decision to perform TAVR was made by a heart team based on the established criteria. Annular sizing was done using either contrast-enhanced computed tomography (CECT) or cardiac magnetic resonance. Patients undergoing valve-in-valve TAVR, or TAVR for an indication other than AS, those with aborted procedures, those who underwent TAVR with a self-expanding valve, and those who underwent annular sizing with transesophageal echocardiography were excluded. Patients with missing important echocardiographic variables and those missing CECT or cardiac magnetic resonance images for annular sizing were also excluded. Patients were then assigned to 1 of 2 groups: patients with a small annulus (annulus area ≤430 mm^2^) and patients with a large annulus (annulus area >430 mm^2^). The cutoff to define a small annulus was similar to the SMART trial.^15^ Four subgroups of patients with an AV area <1 cm^2^ were made according to the flow-gradient pattern: HG AS (AV mean gradient [MG] ≥40 mmHg); C-LFLG AS (MG ​<​40 mmHg), left ventricular ejection fraction (LVEF) ​<50%; paradoxical-LFLG (P-LFLG AS) AS (MG ​< 40 mmHg), stroke volume index (SVI) <35 mL/m^2^, LVEF ≥50%; and normal-flow low-gradient (NFLG) AS (MG ​<40 mmHg), SVI ≥35 mL/m^2^, LVEF ≥50%.^16^ Figure 1 is a Consolidated Standards of Reporting Trials (CONSORT) diagram showing the inclusion and exclusion criteria of the study.

### Echocardiographic Measurements

Patients underwent a transthoracic echocardiogram examination pre-TAVR, before hospital discharge, at 1 to 2 months post-TAVR, at 1 year, 2 years, and 3 years post-TAVR. Echocardiograms were done by operators in accordance with established guidelines.^17^ Echocardiographic parameters included the following variables: AV MG, LVEF, SVI, left ventricular outflow tract velocity time integral, and dimensionless valve index (DVI). Reported LVEF was obtained by the following hierarchical methods: apical biplane Simpson method, apical 2-chamber, apical 4-chamber, and visual estimation. Aortic valve area was calculated according to the continuity equation. The transaortic pressure gradient was calculated from velocity using the simplified Bernoulli equation.

### Annular Size Assessment

Aortic valve dimensions, including the annular diameter and perimeter, were assessed by multidimensional CECT or by cardiac magnetic resonance in the minority of patients with contraindications to CECT (11%). Independent readers evaluated computed tomography images for a full pre-TAVR assessment including the AV annulus, coronary artery origins, cardiac chamber anatomy, and arterial access, using dedicated software (Aquarius iNtuition v4.4, TeraRecon, Foster City, California).

### Outcomes

Primary clinical outcomes were overall mortality and heart failure rehospitalization at 30 days and 3 years post-TAVR. Secondary clinical outcomes included paravalvular leak, ischemic stroke, and permanent pacemaker implantation at 30 days, in addition to major bleeding at 3 years post-TAVR. Clinical outcomes were evaluated according to the updated Valve Academic Research Consortium-3 criteria.^18^ Follow-up time was defined as the time from the procedure to the last documented contact with the patient if they were alive or to the time of documented death. Patients with follow-up times longer than 3 years were censored as alive after 3 years. Valve performance parameters included structural valve deterioration (SVD), hypoattenuated leaflet thickening (HALT), and prosthetic valve thrombosis. Patients with echocardiographic suspicion of HALT on transthoracic echocardiography all underwent a follow-up computed tomography angiography. Therefore, the outcome included findings of HALT on computed tomography angiography, which was reported separately from clinical episodes of prosthetic valve thrombosis. SVD was also evaluated according to the updated Valve Academic Research Consortium-3 criteria.^18^ According to the VARC3, we included patients with intrinsic permanent damage to the prosthetic valve and did not include nonstructural valve dysfunction, thrombosis, or endocarditis. Severe patient-prosthesis mismatch was defined as a predicted^19^ indexed effective orifice area ​≤0.65 cm^2^/m^2^ and moderate patient-prosthesis mismatch was defined as a predicted indexed effective orifice area >0.65 and ​<0.85 cm^2^/m^2^. The main echocardiographic outcomes were LVEF, AV MG, SVI, left ventricular outflow tract velocity time integral, and DVI at each follow-up post-TAVR.

### Statistical Analysis

Statistical analysis was performed with SPSS version 23.0 (IBM Corporation, Armonk, New York). Categorical baseline and procedural characteristics were presented as frequencies (percentage of patients), and continuous variables were presented as mean ​± ​SD with comparisons between the 2 groups using independent sample T-test, chi-square tests, or Fisher exact tests. Clinical outcomes at 3 years were expressed as counts of the first event occurring per patient in the indicated period. For each of the subgroups, clinical outcomes were compared between patients with a small versus large aortic annulus. Clinical outcomes at 30 days were compared between the 2 groups using the chi-square test. Survival analysis comparing the 2 groups was conducted with the Kaplan-Meier method, and statistical comparisons were drawn with the log-rank test. Multivariable Cox proportional hazard modeling adjusted for age, sex, Society of Thoracic Surgeons Predicted Risk of Mortality, and LVEF was used to determine the primary and secondary outcomes; adjusted hazard ratios (HRs) with 95% confidence intervals (CIs) and adjusted *p*-values were reported. *p*-values <0.05 were considered statistically significant.

## Results

### Baseline Characteristics

In the entire study population that included 1866 patients, we identified 709 patients with a small annulus and 1157 patients with a large annulus. Table 1 shows the baseline characteristics of the 2 study populations. The mean age of the population was 79.7 years. Most patients (94%) underwent transfemoral TAVR. Around 80% of patients in the large annulus group were males, and around 80% of patients in the small annulus group were females. Around 87.5% of the patients received an Edwards SAPIEN 3, and 12.5% received an Edwards SAPIEN 3 Ultra (Edwards Lifesciences, Irvine, California). Patients with a small annulus had a lower prevalence of coronary artery disease, atrial fibrillation, and diabetes mellitus and a higher Society of Thoracic Surgeons score.

**Table 1:** Baseline characteristics

Variable	Large annulus N ​= ​1157 (%)	Small annulus N ​= ​709 (%)	*p*-value
General			
Age, y	79.4 ​± ​9.1	80.3 ​± ​8.7	**0.040**
BMI, kg/m^2^	29.1 ​± ​6.4	28.6 ​± ​6.7	0.173
BSA, m^2^	2.01 ​± ​0.26	1.80 ​± ​0.23	**<0.001**
Sex, female	232 (20.1)	564 (79.5)	**<0.001**
Sex, male	925 (79.9)	145 (20.5)	
Race, white	1081 (93.4)	642 (90.6)	**0.036**
Race, black	31 (2.7)	34 (4.8)	
Race, other	45 (3.9)	33 (4.7)	
Flow-gradient pattern			
C-LFLG AS	197 (17.0)	58 (8.2)	**<0.001**
P-LFLG AS	297 (25.7)	220 (31.0)	**0.012**
NFLG AS	74 (6.4)	45 (6.3)	0.967
HG AS	589 (50.9)	386 (54.4)	0.138
History			
CAD	660 (57.0)	311 (43.9)	**<0.001**
Prior MI	277 (23.9)	135 (19.0)	**0.013**
History of AF or atrial flutter	520 (44.9)	262 (37.0)	**0.001**
Diabetes	449 (38.8)	230 (32.4)	**0.006**
Current/recent smoker	62 (5.4)	27 (3.8)	0.127
ESRD	33 (2.9)	25 (3.5)	0.416
Chronic lung disease	481 (41.6)	292 (41.2)	0.869
NYHA 3 or 4	876 (75.7)	545 (76.9)	0.570
Previous PPM	141 (12.2)	61 (8.6)	**0.016**
Previous ICD	43 (3.7)	9 (1.3)	**0.002**
Prior stroke	162 (14.0)	78 (11.0)	0.060
Prior TIA	119 (10.3)	65 (9.2)	0.432
Hypertension	1042 (90.1)	642 (90.6)	0.729
Carotid disease	265 (22.9)	173 (24.4)	0.459
STS-PROM Score	5.5 ​± ​3.9	6.6 ​± ​4.6	**<0.001**
Annulus			
Cross-sectional area, cm^2^	5.3 ​± ​0.7	3.8 ​± ​0.4	**<0.001**
Circumference, cm	8.3 ​± ​0.6	7.1 ​± ​0.5	**<0.001**
Preprocedural			
Hemoglobin, g/dL	12.6 ​± ​1.9	12.1 ​± ​1.8	**<0.001**
Creatinine, mg/dL	1.4 ​± ​1.0	1.2 ​± ​0.8	**<0.001**
Access site			
Transfemoral	1091 (94.3)	660 (93.1)	0.284
Nontransfemoral	66 (5.7)	49 (6.9)	
Valve size (mm)			
>23	1066 (92.1)	87 (12.3)	**<0.001**
≤23	91 (7.9)	622 (87.7)	
Predilation and postdilation			
Predilation	244 (21.1)	150 (21.2)	0.972
Postdilation	387 (33.4)	294 (41.5)	**<0.001**
Postprocedural			
Hemoglobin, g/dL	11.1 ​± ​1.9	10.5 ​± ​2.0	**<0.001**
Creatinine, mg/dL	1.4 ​± ​1.2	1.2 ​± ​1.2	**0.041**

*Notes*. Data presented as n (%) or mean ± standard deviation, where applicable. *p*-values <0.05 were considered statistically significant and are shown in bold.

Abbreviations: AF, ​atrial fibrillation; BMI, body mass index; BSA, body surface area; CAD, ​coronary artery disease; C-LFLG AS, classical low-flow low-gradient aortic stenosis; ESRD, ​end-stage renal disease; HG AS, high-gradient aortic stenosis; ICD, ​implantable cardioverter-defibrillator; MI, ​myocardial infarction; NFLG AS, normal-flow low-gradient aortic stenosis; NYHA, New York Heart Association; P-LFLG AS, paradoxical low-flow low-gradient aortic stenosis; PPM, ​permanent pacemaker; STS-PROM, Society of Thoracic Surgeons Predicted Risk of Mortality; TIA, ​transient ischemic attack.

### Echocardiographic Data

Table 2 summarizes the pre-TAVR and post-TAVR echocardiographic measurements for both groups. The main variables addressed were LVEF, AV area, MG, peak velocity, SVI, and DVI. Before TAVR, there was a statistically significant difference in LVEF between the 2 groups. The mean LVEF of patients in the small annulus group was 59.9%, whereas that of patients in the large annulus group was 54.6%. Patients in the small annulus group also had a higher baseline MG and a lower baseline SVI. Similar findings were noted post-TAVR, where the AV MG and LVEF were also higher in patients with a small annulus compared to those with a large annulus at all follow-up times after TAVR. There was no difference in the incidence of moderate to severe paravalvular leak at discharge.

**Table 2:** Echocardiographic data

Variable	Large annulus N ​= ​1157 (%)	Small annulus N ​= ​709 (%)	*p*-value
Pre-TAVR			
LVEF, %	54.6 ​± ​12.2	59.9 ​± ​10.3	**<0.001**
SVI, mL/m^2^	29.7 ​± ​9.3	27.8 ​± ​9.2	**<0.001**
AV area, cm^2^	0.72 ​± ​0.15	0.69 ​± ​0.16	**<0.001**
AV mean gradient, mmHg	40.6 ​± ​13.6	42.7 ​± ​14.5	**0.002**
AV peak velocity, m/s	4.1 ​± ​0.7	4.2 ​± ​0.7	**<0.001**
DVI	0.22 ​± ​0.05	0.23 ​± ​0.05	**<0.001**
LVOT VTI, mo	0.20 ​± ​0.05	0.23 ​± ​0.06	**<0.001**
Before discharge			
LVEF, %	55.5 ​± ​10.9	59.9 ​± ​8.8	**<0.001**
SVI, mL/m^2^	29.7 ​± ​9.3	26.6 ​± ​8.5	**<0.001**
AV mean gradient, mmHg	9.6 ​± ​4.0	12.1 ​± ​4.8	**<0.001**
DVI	0.53 ​± ​0.13	0.52 ​± ​0.17	0.062
LVOT VTI, mo	0.21 ​± ​0.06	0.24 ​± ​0.06	**<0.001**
Moderate to severe PVL	5 (0.5)	4 (0.6)	0.738
1 mo post-TAVR			
LVEF, %	55.9 ​± ​11.1	59.6 ​± ​9.3	**<0.001**
SVI, mL/m^2^	29.7 ​± ​8.6	26.6 ​± ​7.4	**<0.001**
AV mean gradient, mmHg	10.8 ​± ​3.9	13.9 ​± ​5.1	**<0.001**
DVI	0.49 ​± ​0.11	0.47 ​± ​0.11	**0.037**
LVOT VTI, mo	0.22 ​± ​0.06	0.24 ​± ​0.06	**<0.001**
1-y post-TAVR			
LVEF, %	55.1 ​± ​11.2	60.7 ​± ​9.0	**<0.001**
SVI, mL/m^2^	28.7 ​± ​8.4	25.7 ​± ​7.6	**<0.001**
AV mean gradient, mmHg	11.5 ​± ​5.2	15.2 ​± ​5.6	**<0.001**
DVI	0.47 ​± ​0.11	0.44 ​± ​0.12	**0.009**
LVOT VTI, mo	0.22 ​± ​0.06	0.25 ​± ​0.06	**<0.001**
2-y post-TAVR			
LVEF, %	55.3 ​± ​12.2	60.9 ​± ​8.4	**<0.001**
SVI, mL/m^2^	28.1 ​± ​8.0	26.3 ​± ​8.2	**0.014**
AV mean gradient, mmHg	11.1 ​± ​4.6	14.9 ​± ​6.0	**<0.001**
DVI	0.47 ​± ​0.11	0.43 ​± ​0.10	**0.002**
LVOT VTI, mo	0.22 ​± ​0.06	0.24 ​± ​0.07	**<0.001**
3-y post-TAVR			
LVEF, %	55.3 ​± ​12.5	60.6 ​± ​8.7	**<0.001**
SVI, mL/m^2^	28.9 ​± ​8.1	25.7 ​± ​7.9	**<0.001**
AV mean gradient, mmHg	11.1 ​± ​5.0	14.9 ​± ​6.6	**<0.001**
DVI	0.46 ​± ​0.12	0.44 ​± ​0.11	**0.043**
LVOT VTI, mo	0.21 ​± ​0.06	0.24 ​± ​0.07	**<0.001**

Data presented as n (%) or mean ± standard deviation, where applicable. *p*-values <0.05 were considered statistically significant and are shown in bold.

Abbreviations: AV, ​aortic valve; DVI, ​dimensionless valve index; LVEF, ​left ventricular ejection fraction; LVOT, ​left ventricular outflow tract; PVL, ​paravalvular leak; SVI, ​stroke volume index; TAVR, transcatheter aortic valve replacement; VTI, ​velocity time integral.

### Procedural and Clinical Outcomes for the Overall Small Annulus vs. Large Annulus Population

Table 3 summarizes the in-hospital procedural outcomes, in addition to HALT, SVD, and patient-prosthesis mismatch. The overall incidence of severe patient-prosthesis mismatch, SVD, and the incidence of late HALT were higher in the small annulus population. There was no difference in procedural outcomes including ischemic stroke and AV reintervention between the 2 groups.

**Table 3:** SVD, HALT, patient prosthesis mismatch, and procedural outcomes

Variable	Large annulus N ​= ​1157 (%)	Small annulus N ​= ​709 (%)	*p*-value
SVD			
Overall	7 (0.6)	11 (1.6)	**0.042**
Mean time to SVD (mo)	39.9 ​± ​18.2	45.8 ​± ​21.9	0.565
HALT and thrombosis			
Overall	8 (0.7)	17 (2.4)	**0.002**
During 30 d	1 (0.1)	0 (0)	1.000
During 3 mo	5 (0.4)	1 (0.1)	0.418
During 1 y	6 (0.5)	6 (0.8)	0.389
During 2 y	6 (0.5)	12 (1.7)	**0.012**
During 3 y	7 (0.6)	15 (2.1)	**0.003**
Prosthetic valve thrombosis	3 (0.3)	4 (0.6)	0.438
Patient prosthesis mismatch			
Moderate	416 (36.0)	419 (59.1)	**<0.001**
Severe	11 (1.0)	31 (4.4)	**<0.001**
In-hospital events			
Ischemic stroke	10 (0.9)	4 (0.6)	0.586
Hemorrhagic stroke	0 (0)	0 (0)	-
AV reintervention	3 (0.3)	0 (0)	0.293
Coronary compression/obstruction	3 (0.3)	0 (0)	0.293
PCI	11 (1.0)	7 (1.0)	0.937

Data presented as n (%) or mean ± standard deviation, where applicable. *p*-values <0.05 were considered statistically significant and are shown in bold.

Abbreviations: AV, ​aortic valve; HALT, hypoattenuated leaflet thickening; PCI, percutaneous coronary intervention; SVD, structural valve deterioration.

Table 4 shows the 30-day outcomes of both populations. There was no difference in 30-day mortality, heart failure rehospitalization, ischemic stroke, or permanent pacemaker implantation between small and large annulus patients.

**Table 4:** Outcomes at 30 days

Variable	Large annulus N ​= ​1157 (%)	Small annulus N ​= ​709 (%)	*p*-value
30 d			
Mortality	10 (0.9)	5 (0.7)	0.709
Heart failure rehospitalization	14 (1.2)	12 (1.7)	0.388
Acute myocardial infarction	3 (0.3)	0 (0)	0.293
Major bleeds	18 (1.6)	14 (2.0)	0.499
Ischemic stroke	13 (1.1)	6 (0.8)	0.562
Permanent pacemaker implantation	75 (6.5)	44 (6.2)	0.813

Data presented as n (%).

### Clinical Outcomes for the Overall Population Across the Flow-Gradient Patterns

**Figure 2:**
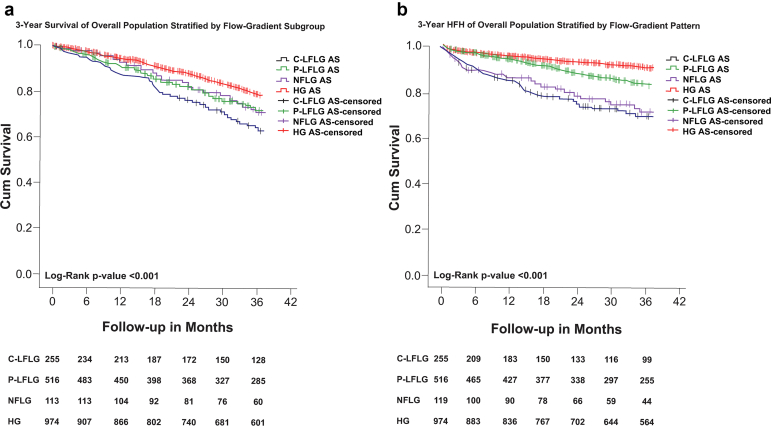
Kaplan-Meier showing (a) the 3-year overall survival of the population stratified by flow-gradient pattern and (b) the 3-year HFH of the population stratified by flow-gradient pattern. Abbreviations: C-LFLG AS, classical low-flow low-gradient aortic stenosis; HFH, heart failure rehospitalization; HG AS, high-gradient aortic stenosis; NFLG AS, normal-flow low-gradient aortic stenosis; P-LFLG AS, paradoxical low-flow low-gradient aortic stenosis.

Out of 1866 patients (57.6%), 1074 had at least 3 years of follow-up. The median time to follow-up was 3.3 years. Figure 2 shows the Kaplan-Meier plots for mortality and heart failure rehospitalization for the overall population, stratified by flow-gradient patterns. Patients with C-LFLG AS had the highest risk of mortality and heart failure rehospitalization.

### Clinical Outcomes for Small vs. Large Annulus Across the Flow-Gradient Patterns

**Figure 3:**
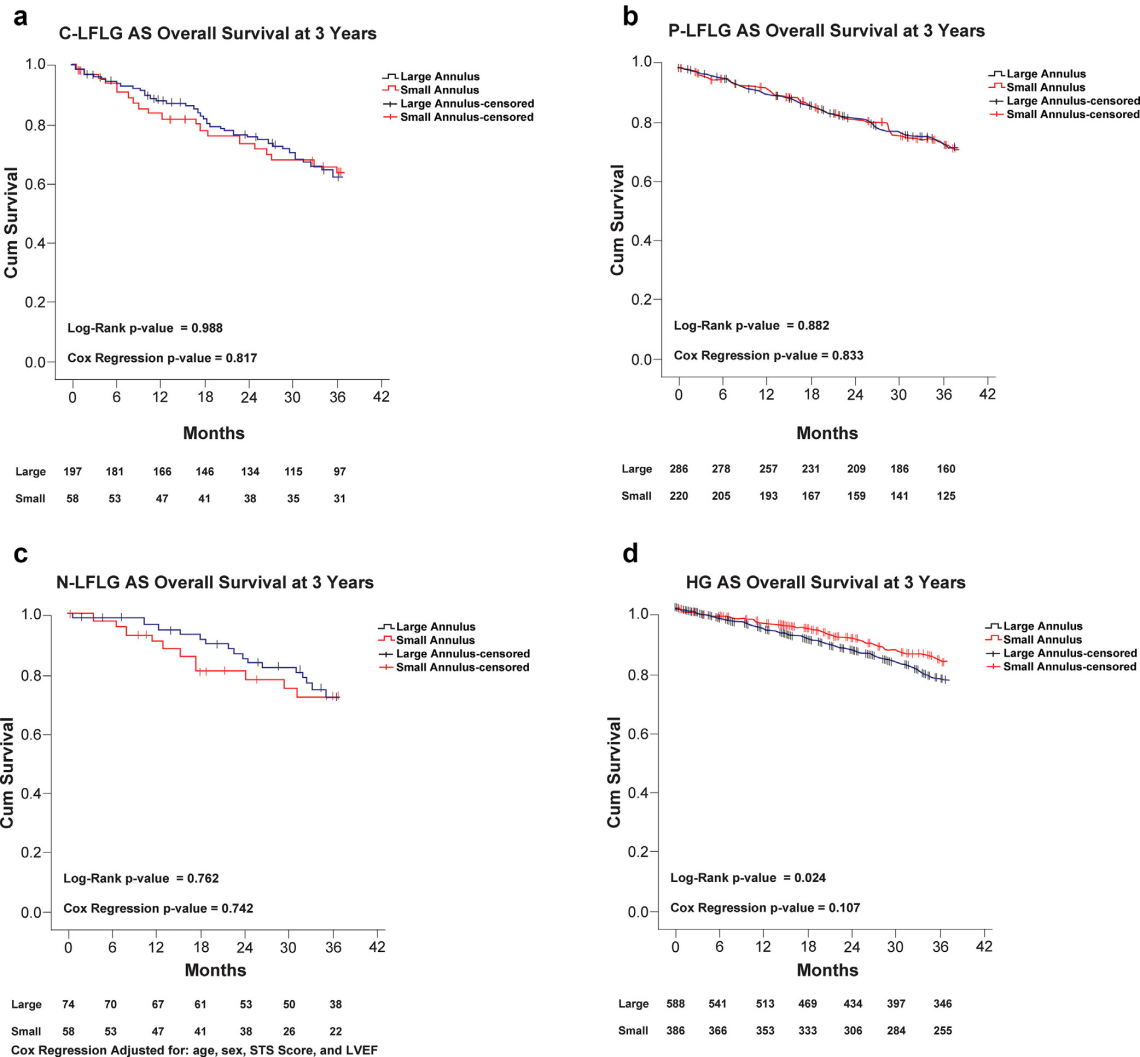
Kaplan-Meier plots for overall survival at 3 years for small vs. large annulus in patients with (a) C-LFLG AS, (b) P-LFLG AS, (c) NFLG AS, and (d) HG AS. Abbreviations: C-LFLG AS, classical low-flow low-gradient aortic stenosis; HG AS, high-gradient aortic stenosis; LVEF, left ventricular ejection fraction; NFLG AS, normal-flow low-gradient aortic stenosis; P-LFLG AS, paradoxical low-flow low-gradient aortic stenosis; STS, Society of Thoracic Surgeons.

**Figure 4:**
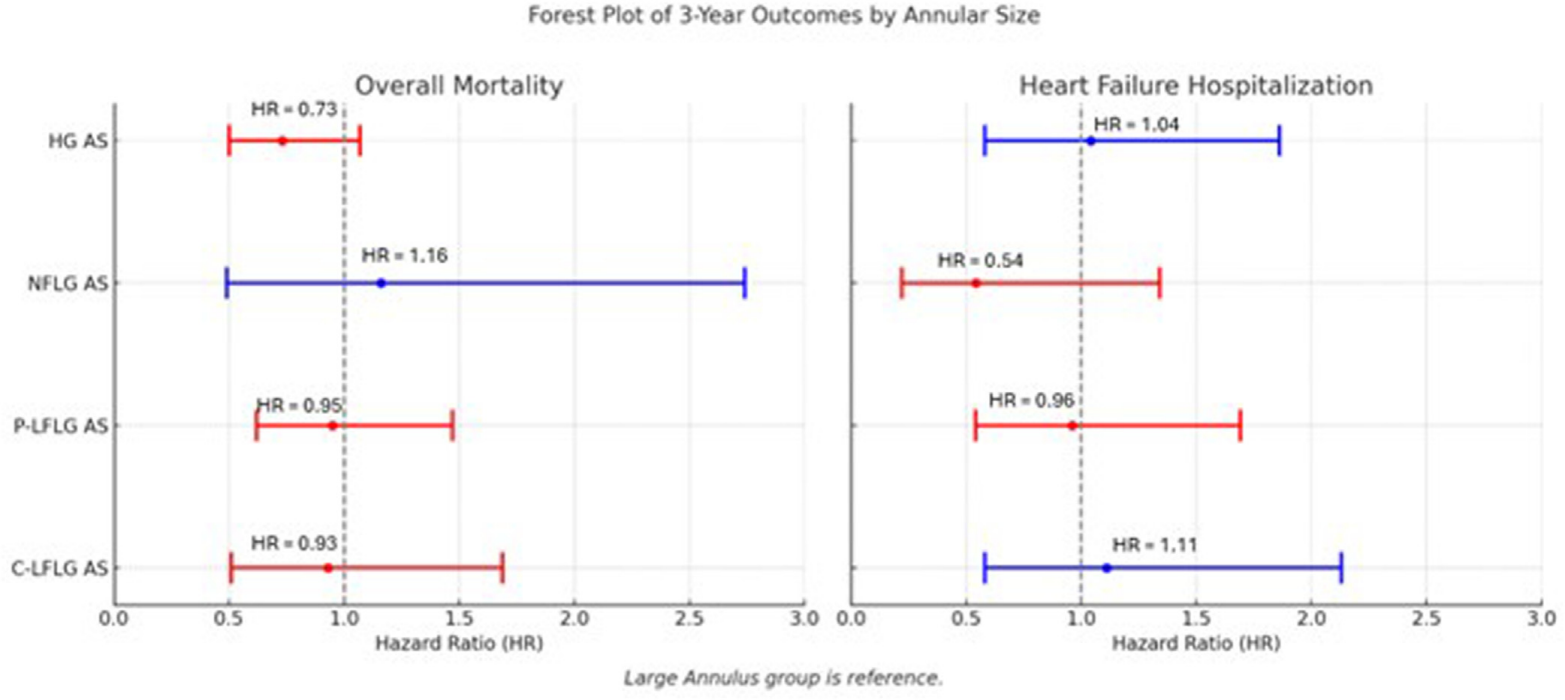
Forest plot showing the point estimates for the small annulus group compared to the large annulus group, stratified by flow-gradient pattern. Abbreviations: C-LFLG AS, classical low-flow low-gradient aortic stenosis; HG AS, high-gradient aortic stenosis; HR, hazard ratio; NFLG AS, normal-flow low-gradient aortic stenosis; P-LFLG AS, paradoxical low-flow low-gradient aortic stenosis.

**Supplementary Figure 1:**
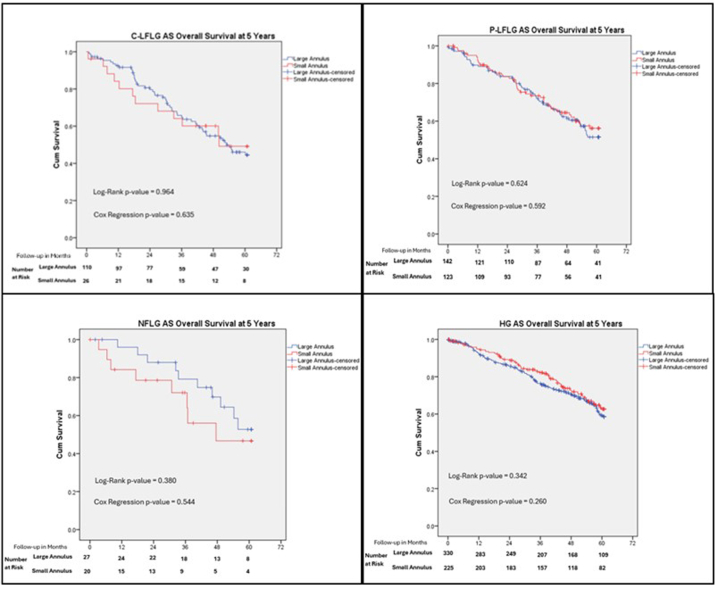
Overall survival at 5 years for patients undergoing TAVR between 2016-2018.

Table 5 shows the results of the Cox proportional hazard models comparing 3-year mortality, heart failure rehospitalization, and major bleeding events between patients with small and large annuli in the C-LFLG, P-LFLG, NFLG, and HG AS groups separately. There were no significant differences in any of the clinical outcomes between the small and large annulus groups in any of the flow gradient patterns. Figure 3 shows the 4 Kaplan-Meier plots for mortality at 3 years in each flow-gradient subgroup separately. Figure 4 is a forest plot showing the point estimates for mortality and heart failure rehospitalization of the small annulus group compared to the large annulus group, stratified by flow-gradient pattern. Supplementary Figure 1 shows the 4 Kaplan-Meier plots for mortality at 3 years in each flow-gradient subgroup separately.

**Table 5:** Results of primary outcomes using Cox proportional hazards using large annulus as a reference stratified by flow-gradient pattern

3 y outcomes	Adjusted HR	95% CI	*p*-value
C-LFLG AS			
Overall mortality	0.93	0.51-1.69	0.817
Heart failure hospitalization	1.11	0.58-2.13	0.746
Bleeding	1.37	0.54-3.47	0.510
P-LFLG AS			
Overall mortality	0.95	0.62-1.47	0.833
Heart failure hospitalization	0.96	0.54-1.69	0.875
Bleeding	1.71	0.83-3.52	0.143
NFLG AS			
Overall mortality	1.16	0.49-2.74	0.742
Heart failure hospitalization	0.54	0.22-1.34	0.180
Bleeding	0.59	0.19-1.83	0.364
HG AS			
Overall mortality	0.73	0.50-1.07	0.107
Heart failure hospitalization	1.04	0.58-1.86	0.907
Bleeding	0.72	0.41-1.27	0.250

*Notes.* Adjusted for age, sex, STS risk score, and LVEF.

A large annulus group is referenced.

Abbreviations: C-LFLG AS, classical low-flow low-gradient aortic stenosis; CI, confidence interval; HG AS, high-gradient aortic stenosis; HR, hazard ratio; LVEF, ​left ventricular ejection fraction; NFLG AS, normal-flow low-gradient aortic stenosis; P-LFLG AS, paradoxical low-flow low-gradient aortic stenosis; STS, Society of Thoracic Surgeons.

Table 6 shows the effect of baseline SVI (tertiles and continuous using Cox regression) on 3-year mortality in patients with a small annulus and large annulus separately. SVI (continuous or tertiles) did not affect the 3-year mortality rate in the small or large annulus populations.

**Table 6:** The effect of baseline SVI (tertiles and continuous using Cox regression) on 3-year mortality in patients with a small annulus and large annulus separately

3 y mortality	Adjusted HR	95% CI	*p*-value
Small annulus			
Baseline SVI tertile 3 vs. 1	0.81	0.54-1.22	0.308
Baseline SVI continuous	0.98	0.97-1.01	0.135
Large annulus			
Baseline SVI tertile 3 vs. 1	1.03	0.76-1.40	0.853
Baseline SVI continuous	1.00	0.99-1.02	0.819

Adjusted for age, sex, STS risk score, and LVEF.

Abbreviations: CI, confidence interval; HR, hazard ratio; LVEF, ​left ventricular ejection fraction; STS, Society of Thoracic Surgeons; SVI, stroke volume index.

### Predictors of 3-Year Mortality

Table 7 shows the results of the Cox regression model exploring the predictors of 3-year mortality in the overall population. C-LFLG AS and P-LFLG AS, end-stage renal disease (ESRD), chronic lung disease, a history of atrial fibrillation, and anemia, all significantly correlated with a higher mortality rate, whereas a small aortic annulus, C-LFLG AS, P-LFLG AS, and NFLG AS were not predictors of mortality at 3 years post-TAVR.

**Table 7:** Results of the Cox regression model for the predictors of 3-year mortality in the overall population

Predictors of 3-y mortality in overall population			
Variable	HR	95% CI	*p*-value
Small annulus	0.91	0.68-1.20	0.491
C-LFLG AS (vs. HG AS)	**1.38**	1.01-1.89	**0.049**
P-LFLG AS (vs. HG AS)	**1.36**	1.05-1.77	**0.018**
NFLG AS (vs. HG AS)	0.89	0.56-1.42	0.628
Age	1.01	0.99-1.03	0.118
Sex, female sex	0.72	0.51-1.03	0.073
Race, black race	0.85	0.50-1.46	0.559
BMI	1.01	0.97-1.04	0.846
BSA	0.66	0.27-1.62	0.360
CAD	0.91	0.70-1.18	0.466
Prior MI	0.86	0.63-1.16	0.318
Prior stroke	1.18	0.88-1.59	0.270
Diabetes mellitus	1.10	0.87-1.37	0.462
Current or recent smoking	1.48	0.93-2.33	0.095
ESRD	**3.95**	2.56-6.10	**<0.001**
Chronic lung disease	**1.34**	1.06-1.68	**0.013**
History of AF or Aflutter	**1.52**	1.20-1.92	**0.001**
Prior pacemaker	0.95	0.68-1.34	0.788
Nontransfemoral access	1.34	0.91-1.95	0.136
Hemoglobin pre-TAVR	**0.80**	0.75-0.85	**<0.001**
Discharge mean gradient	0.98	0.96-1.01	0.301
Moderate to severe discharge AR	1.97	0.61-6.31	0.256
Moderate to severe discharge MR	1.26	0.89-1.76	0.189
Moderate to severe discharge TR	1.13	0.84-1.53	0.426

*Notes.p*-values <0.05 were considered statistically significant and are shown in bold.

Abbreviations: AF, ​atrial fibrillation; Aflutter, atrial flutter; AR, ​aortic regurgitation; BMI, body mass index; BSA, body surface area; CAD, ​coronary artery disease; C-LFLG AS, classical low-flow low-gradient aortic stenosis; CI, confidence interval; ESRD, ​end-stage renal disease; HG AS, high-gradient aortic stenosis; HR, hazard ratio; MI, ​myocardial infarction; MR, ​mitral regurgitation; NFLG AS, normal-flow low-gradient aortic stenosis; P-LFLG AS, paradoxical low-flow low-gradient aortic stenosis; TAVR, transcatheter aortic valve replacement; TR, ​tricuspid regurgitation.

### Impact of Severe Patient-Prosthesis Mismatch on Mortality in Patients With a Small Annulus

Table 8 shows the results of the univariate Cox regression models showing the impact of severe patient-prosthesis mismatch on the mortality of patients with a small annulus across flow-gradient patterns. Severe patient-prosthesis mismatch did not significantly increase the risk of mortality in patients with small annuli in any of the flow-gradient subgroups.

**Table 8:** Results of the univariate Cox regression models showing the impact of severe patient-prosthesis mismatch on the mortality of patients with a small annulus across flow-gradient patterns

Patient with small annuli			
3 y mortality	HR	95% CI	*p*-value
C-LFLG AS			
Severe patient-prosthesis mismatch	1.21	0.16-9.08	0.853
P-LFLG AS			
Severe patient-prosthesis mismatch	1.51	0.47-4.82	0.490
NFLG AS			
Severe patient-prosthesis mismatch	0.05	0.01-1069.51	0.545
HG AS			
Severe patient-prosthesis mismatch	0.05	0.01-7.79	0.240

Abbreviations: C-LFLG AS, classical low-flow low-gradient aortic stenosis; CI, confidence interval; HG AS, high-gradient aortic stenosis; HR, hazard ratio; NFLG AS, normal-flow low-gradient aortic stenosis; P-LFLG AS, paradoxical low-flow low-gradient aortic stenosis.

## Discussion

In the present study, we focused on comparing the outcomes of patients with a small vs. large aortic annulus across the different flow-gradient patterns of AS after TAVR. The key findings were as follows:

1. Thirty-eight percent of patients in this study had small annuli, 80% of which were women.
2. More patients with a large annulus had C-LFLG AS, whereas more patients with a small annulus had P-LFLG AS.
3. Annular size was not a predictor of overall mortality, whereas C-LFLG AS and P-LFLG AS, ESRD, atrial fibrillation, chronic lung disease, and anemia were significant predictors of mortality in the overall population.
4. Patients with a smaller aortic annulus have higher AV mean gradients, lower DVI, and a higher incidence of SVD and HALT post-TAVR compared to those with a large annulus.
5. There is no difference in clinical outcomes between patients with a small compared to large annulus in any of the 4 subgroups: C-LFLG AS, P-LFLG AS, NFLG AS, and HG AS.

Multiple risk factors and echocardiographic parameters correlate with poor outcomes post-TAVR. In the present study, we found that LFLG AS (classical and paradoxical), ESRD, atrial fibrillation, chronic lung disease, and anemia were significant predictors of mortality. This agrees with the literature where studies found ESRD,^20^ anemia,^21^ atrial fibrillation,^22^ and chronic lung disease^23^ to correlate with mortality post-TAVR.

In the present study, we also found that patients with C-LFLG AS had a higher risk of mortality compared to those with HG AS and that C-LFLG AS was more prevalent in the large annulus group, whereas P-LFLG AS was more prevalent in the small annulus group in our study population. Previous studies also showed a difference in mortality rates after TAVR in patients with different flow-gradient patterns of AS. Patients with C-LFLG AS had the highest rates of mortality,^3^^,^^4^^,^^6^^,^^8^ and those with P-LFLG AS had similar mortality rates when compared to HG AS.^24^

In the literature, patients with a small annulus comprise 22% to 44% of the overall population,^25^^,^^26^ the majority of whom are women.^11^^,^^27^ In the present study, we used an annulus area ≤430 mm^2^ to define a small annulus, similar to the SMART trial.^15^ Using this definition, 37% of the patients in the present study had a small annulus. Similarly to the SMART trial, the mean age of that population in our study was 80 years. In addition, women comprised 80% of the patients with a small annulus, a finding that agrees with the literature.^12^^,^^15^^,^^28^, ^29^, ^30^

Previous studies on surgical AV replacement with long-term follow-up demonstrated that smaller valve sizes and higher AV MG at discharge correlate with worse survival and less valve durability.^1^^,^^2^ It is still a matter of debate whether this is true for patients who underwent TAVR. Previous studies showed mixed results regarding clinical outcomes and valve hemodynamics post-TAVR in patients with different aortic annular sizes. Analyses from the WIN-TAVI registry and the Core Valve US Pivotal Trial showed that annular size did not impact the mortality rate in patients who underwent TAVR.^9^^,^^10^ On the other hand, an analysis of the PARTNER Trial concluded that patients with a large annulus had a higher mortality rate compared to those with a small or intermediate annulus.^31^

In the present study, we found that annular size did not have an impact on clinical outcomes. Patients with a small annulus were not found to have a higher mortality risk compared to those with a large annulus in the overall population and each of the flow-gradient subgroups separately. Additionally, we found that SVI had no impact on clinical outcomes in patients with small and large annuli separately. This might be due to chance or because the majority of the LFLG AS population had P-LFLG AS, with normal left ventricular systolic function and a lower risk of mortality.^24^ No previous studies examined the impact of both annular size and flow-gradient subgroup on post-TAVR clinical outcomes.

All-cause mortality, especially in the elderly population of patients undergoing TAVR, can be affected by multiple factors that are unrelated to the TAVR valve type or annular size. Therefore, it is important to differentiate clinical outcomes from valve performance parameters. We found that patients with a small annulus have worse hemodynamic performance and higher rates of SVD, severe patient-prosthesis mismatch, and HALT post-TAVR compared to those with a large annulus. However, the present study is limited by the intermediate-term follow-up of 3 years (and 5 years in patients who underwent TAVR between 2016 and 2018 [Supplementary Figure 1]). Longer follow-up might reveal differences in clinical outcomes between patients with small and large annulus.

A thorough pre-TAVR assessment that includes multimodality imaging to understand the patient’s risk profile, anatomic profile, annular size, and flow-gradient pattern is essential, as it provides information that could help guide the choice of the valve and implantation technique. Given that one-third of patients in TAVR clinical trials have a small annulus, with the majority being women, it is important to follow these patients up and understand their hemodynamic profile, which could be worse than those with a large annulus. It is also important to understand the specific risk factors and flow-gradient patterns for each patient to better stratify patients with AS based on their risk. This will be beneficial for better management of patients referred for TAVR.

The following limitations should be noted in the present study. First, this was a single-center retrospective, nonrandomized study, which could be subject to all the biases inherent in retrospective analyses, including the presence of residual confounders even after adjustment. Second, the present study was conducted at a single high-volume referral center and thus could be subject to selection bias. Third, the patients in this study are symptomatic undergoing TAVR. It is possible that there are many asymptomatic patients who are not referred for TAVR, and they may have different characteristics and outcomes. Lastly, a small number of patients were excluded because they missed important pre-TAVR echocardiographic measurements, which could potentially introduce selection bias. However, the number of patients excluded was small relative to the whole population.

In summary, among patients with AS undergoing TAVR with a balloon-expandable valve, those with a small annulus had similar clinical outcomes to those with a large annulus in all flow-gradient subgroups (C-LFLG, P-LFLG, NFLG, and HG AS), suggesting no impact of the annular size on outcome. Patients with small annuli had worse hemodynamic performance at all follow-up intervals.

## Ethics Statement

The research reported has adhered to the relevant ethical guidelines.

## Funding

The authors have no funding to report.

## Disclosure Statement

The authors report no conflict of interest.

## Review Statement

Full responsibility for the editorial process for this article was delegated to Jeffrey Popma, MD.
